# Unleashing the potential of eHealth in outpatient cancer care for patients undergoing immunotherapy—a quantitative study considering patients’ needs and current healthcare challenges

**DOI:** 10.3389/fdgth.2024.1414442

**Published:** 2024-10-21

**Authors:** Tobias A. W. Holderried, Isabel Stasik, Marie-Therese Schmitz, Friederike Schmitz, Tizian K. Meyer, Leonie Stauß, Martin Kirschner, Dirk Skowasch, Jennifer Landsberg, Matthias Schmid, Peter Brossart, Martin Holderried

**Affiliations:** ^1^ Department of Oncology, Hematology, Immuno-Oncology and Rheumatology, University Hospital Bonn, Bonn, Germany; ^2^ Center for Integrated Oncology (CIO) ABCD, Aachen Bonn Cologne Düsseldorf, Germany; ^3^ Department of Ophthalmology, University Hospital Bonn, Bonn, Germany; ^4^ Institute for Medical Biometry, Informatics and Epidemiology (IMBIE), University Hospital Bonn, Bonn, Germany; ^5^ Department of Internal Medicine, Waldkrankenhaus Bonn, Bonn, Germany; ^6^ Department of Medical Strategy, Process- and Quality Management, Tuebingen University Hospital, Tuebingen, Germany; ^7^ Institute of Health Care and Public Management, University of Hohenheim, Stuttgart, Germany; ^8^ Department of Hematology, Oncology, Hemostaseology and Stem Cell Transplantation, RWTH Aachen University, Aachen, Germany; ^9^ Department of Medicine II, University Hospital Bonn, Bonn, Germany; ^10^ Department of Dermatology, University Hospital Bonn, Bonn, Germany; ^11^ eHealth Research Group, Department of Otolaryngology—Head and Neck Surgery, Tuebingen University Hospital, Tuebingen, Germany

**Keywords:** immunotherapy, immune checkpoint inhibitors, cellular therapy, outpatient care, eHealth, telemedicine, digital health

## Abstract

**Background:**

The use of online information and communication is globally increasing in the healthcare sector. In addition to known benefits in other medical fields, possible specific potentials of eHealth lie in the monitoring of oncological patients undergoing outpatient therapy. Specifically, the treatment with immune checkpoint inhibitors (ICI) requires intensive monitoring due to various possible negative side effects. The present study explores cancer patients’ perspectives on eHealth and demonstrates how eHealth applications, from the patients’ point of view, can contribute to further improving outpatient immunotherapy.

**Methods and findings:**

Our multicenter study was executed at the university hospitals in Bonn and Aachen. A structured questionnaire was distributed to patients receiving outpatient immunotherapy. Contents addressed were (1) the patients’ attitude towards eHealth applications, (2) the use of modern information and communications technologies (ICT) in (2a) everyday life and (2b) health-related information search including eHealth literacy, (3) the use of internet-enabled devices as well as (4) socio-demographic data. 164 patients were included in the study, of whom 39.0% were female and 61.0% male and the average age was 62.8 years. Overall, there was a high distribution of internet-enabled devices for everyday use and a great interest in integrating eHealth applications into outpatient immunotherapy. The assessment of eHealth potentials significantly depended on age. The younger participants demonstrated a broader use of modern ICT and a higher affinity for its use in outpatient immunotherapy. In some aspects, level of education and gender were also relevant factors influencing the patients’ view on eHealth.

**Conclusion:**

This study demonstrates the potential for further integration of eHealth applications into outpatient immunotherapy from the patients’ perspective. It indicates a dependency on age and educational level for the further integration of eHealth into patient care in oncology. Due to particular patient needs regarding age, level of education, gender and other subgroups, specific education and training as well as target-group specific digital health interventions are necessary to fully utilize the potentials of eHealth for outpatient immunotherapy. Future studies are required to specifically address target-group specific usability of eHealth applications and eHealth literacy, as well as to address information security and data protection.

## 1 Introduction

The use of eHealth is already strongly implemented in in- and outpatient care and represents an emerging field in scientific research (1). By WHO-definition, “eHealth” is a collective term for “the cost-effective and secure use of information and communication technologies in support of health and health-related fields” (2). An increasing number of scientific studies from various medical disciplines in different countries discuss both the potential and impact of eHealth on patient care and on organizational processes in the healthcare sector (3–14). Highlighting this increasing interest, a Pubmed search for “eHealth” currently yields >68,000 results (03/2024). Considering the research numbers at the time of our study, this represents an annual growth rate of approximately 27.08% (15). Essential components of eHealth are the digital care processes and the technologies used, whose development is rapidly progressing. Previous studies suggest that eHealth has the potential to promote healthcare, particularly by enhancing the quality, efficiency and efficacy of patient care (16, 17). Several pilot studies have also been conducted in the field of oncology regarding the potential use of eHealth applications, confirming the experiences gained in other medical areas (18–20). Of note is the enhanced interdisciplinary collaboration, the alleviation of burdens on specialists, and the mitigation of the continuously escalating expenses within the healthcare system. A primary objective of teleoncology is to facilitate patients’ access to treatment in close proximity to their homes within familiar surroundings, while minimizing inconveniences such as lengthy waiting periods, extensive travel, or exposure to infectious agents in waiting areas. This objective is realized through various digital applications, including online consultations, tools for side effect monitoring and online monitoring in real-time and remote, among others. However, alongside the potential benefits, issues such as data security, technological expenses, and the need for training and continuous education remain subject of ongoing debate (21).

Of particular interest for the use of eHealth are cancer patients undergoing immunotherapy, especially the predominantly outpatient treatments with immune checkpoint inhibitors (ICI). These are used in the treatment of a variety of malignancies (22) and can often be carried out on an outpatient basis (23). In some cases, however, these therapies can lead to severe autoimmune processes that require prompt treatment (23). Specific monitoring is therefore necessary during outpatient therapy with ICI, ideally being further supported by suitable eHealth applications in the future. Therefore, a comprehensive assessment of the potentials of eHealth from the perspective of patients undergoing immunotherapy with ICI could provide further important information for the targeted development of eHealth applications (24–26). To this end, we addressed the following aspects in the current study in this specific patient population: (1) the patients’ attitude towards eHealth applications, (2) the use of modern information and communications technologies (ICT) in (2a) everyday life and (2b) health-related information search including eHealth literacy, (3) the use of internet-enabled devices as well as (4) socio-demographic data.

## 2 Methods

### 2.1 Design

This multicenter prospective study analysed a structured questionnaire taken at two university hospitals in Germany. The survey was conducted from September 2019 to March 2021. For safety, the survey was suspended for 5 months at the start of the Covid-19 pandemic (March 2020–August 2020).

### 2.2 Ethics

This questionnaire-based study was audited by the IRB (Institutional Ethics Committee of the Medical Faculty and University Hospital Bonn, approval number 385/19). All patients participated voluntarily and gave their informed consent.

### 2.3 Study population

The survey was conducted at the University Hospital Bonn, Germany and the University Hospital Aachen, Germany. During the time the study was conducted, all participants received outpatient therapy with ICI or outpatient therapy with ICI in combination with chemotherapy. The reason for treatment was cancer. To participate in the survey, patients had to be at least 18 years old and in a good cognitive condition. To assess the cognitive condition of the study participants, we evaluated their medical history in a personal conversation, reviewed the medical documents for conditions that might impair cognitive function, and checked the current medications with special attention to those that could affect cognitive performance. The European Health Council defines an “older person” as someone aged 65 and over (27). Previous studies in other medical fields showed a reluctance of elderly people towards telemedicine (28–30). In our study cohort bronchial carcinoma was the most common cancer (39.9%) with the mean age of onset being approximately 65 years. Therefore, we divided the study population for age-specific analysis purposes into 2 age groups (under 65 years vs. 65 years and older).

### 2.4 Questionnaire

Our questionnaire was developed by an interdisciplinary team of physicians specialized in hematology and oncology, eHealth specialists, quality managers, and public health researchers, and based on current literature and experiences in various medical fields (31–36). To avoid bias in the results due to a focus on digitally savvy individuals, the anonymous study was conducted using a paper-based questionnaire rather than a digital online questionnaire. The paper-based structured questionnaire included the following aspects: (1) the patients’ attitude towards eHealth applications, (2) the use of modern ICT in (2a) everyday life and (2b) health-related information search including eHealth literacy, (3) the use of internet-enabled devices and (4) socio-demographic data of this specific patient population. The questionnaire comprised close-ended questions (e.g., smartwatch ownership, health data recording) and rating scales for specific measures (e.g., improvement in treatment quality through online communication). The 8-item-based eHealth Literacy Scale (eHEALS) was included in the questionnaire to assess the perceived knowledge and skills for using digital information technology for health purposes among the study population (37–40). In addition to age, we divided the patient cohort into different educational levels. Here we used the ISCED (International Standard Classification of Education), an international system developed by UNESCO for classifying and comparing educational programs and qualifications. The ISCED scale is used to categorize educational programs and qualifications in a uniform way to enable international comparisons. ISCED consists of different levels that reflect the different levels of education. For the purpose of simplicity, we summarized the groups in 0–2, 3–4, and 5–8. The educational levels of the individual stages are as follows: ISCED (0) Early Childhood Education, ISCED (1) Primary education, ISCED (2) Lower-secondary education, ISCED (3) Upper-secondary education, ISCED (4) Post-secondary non-tertiary education, ISCED (5) Short-cycle tertiary education, ISCED (6) Bachelor's or equivalent level, ISCED (7) Master's or equivalent level, ISCED (8) Doctoral or equivalent level (41).

### 2.5 Statistical analysis

For an initial overview of the collected data, we conducted a descriptive analysis. Bivariate analyses were employed to examine the relationships between the sociodemographic aspects of the study population, the current use of modern digital media, the type of tumor, and the attitude towards eHealth applications for further use in cross-sectoral care. To detect statistically significant trends in the queried aspects, the surveyed statements regarding the potential of eHealth were transformed from a 4-point Likert scale into binary response variables. “Fully” and “fairly” were considered positive, while “not at all” and “rather not” were considered negative. The same procedure was applied to the 5-point Likert responses for the eHealth Literacy Scale to create the dataset for these bivariate statistical calculations, the statements were transformed into the following response variables: positive (fully, fairly), negative (rather not, not at all), and neutral (do not know). Cross-tabulation and Pearson's chi-square tests were used to evaluate the differences in relative frequencies between age groups and levels of education. The study-specific results were presented as numbers and percentages for valid cases, and two-tailed *p*-values. To examine the association between age, gender, education level, community size and type of therapy, and internet usage for daily life activities or for health reasons, we employed multiple logistic regression. Associations between age, gender, educational level and attitudes towards various eHealth usages were analyzed using multinomial logistic regression. The results are expressed as odds ratios (ORs) with 95% confidence intervals (CIs). Low and medium levels of education were pooled for the regression analyses. Participants who did not answer specific questions were excluded from the analyses of those questions. Missing data for each individual question evaluated never exceeded 15% and therefore did not significantly affect the interpretation of the results. For all analyses conducted in our study, *p*-values < 0.05 were considered statistically significant. R version 4.3.1 (42) was used for all statistical analyses.

## 3 Results

### 3.1 Characteristics of the study sample

208 patients undergoing outpatient immunotherapy with ICI were asked for participation and 164 evaluable questionnaires were returned corresponding to a participation rate of 78.8%. This provides an overall good basis for the statistical analyses conducted in the study. The average age of the patients was 62.8 years (SD 10.9 years) and the female-to-male ratio was 0.64–1. Of the surveyed patients, 85.6% received ICI alone while 14.4% were treated with a combination of ICI and chemotherapy. 51.7% of the patients had been in therapy for 0–6 months, while 48.3% had a current therapy duration of more than 6 months. The main tumors treated were bronchial carcinoma (39.9%), malignant melanoma (22.2%), urological malignancies (18.3%) as well as head and neck tumors (13.7%). An overview of additional sociodemographic factors and age-related patient characteristics can be found in Table 1.

**Table 1:** Age-related characteristics of the study sample (*n* = 164).

	Total (*n* = 164)	Age < 65 years (*n* = 89)	Age ≥ 65 years (*n* = 75)	*p*-value
	*n* (%)	*n* (%)	*n* (%)	
Gender				
Female	62 (39.0)	37 (42.5)	25 (34.7)	
Male	97 (61.0)	50 (57.5)	47 (65.3)	n.s. (0.400)
ISCED				
Low (level 0–2)	14 (8.9)	7 (8.0)	7 (10.0)	
Medium (level 3–4)	101 (64.3)	57 (65.5)	44 (62.9)	
High (level 5–8)	42 (26.8)	23 (26.4)	19 (27.1)	n.s. (0.898)
Community size (population)				
<2.000	33 (22.0)	18 (20.7)	15 (23.8)	
2.001–30.000	63 (42.0)	38 (43.7)	25 (39.7)	
>30.000	54 (36.0)	31 (35.6)	23 (36.5)	n.s. (0.857)
Cancer type				
Bronchial carcinoma	61 (39.9)	32 (36.8)	29 (43.9)	
Malignant melanoma	34 (22.2)	22 (25.3)	12 (18.2)	
Urological tumor	28 (18.3)	18 (20.7)	10 (15.2)	
Tumor in the head/neck area	21 (13.7)	11 (12.6)	10 (15.2)	
Other	9 (5.9)	4 (4.6)	5 (7.6)	n.s. (0.448)
Type of therapy				
Immunotherapy	131 (85.6)	75 (86.2)	56 (84.8)	
Combination with chemotherapy	22 (14.4)	12 (13.8)	10 (15.2)	n.s. (0.996)
Travel time to university hospital				
<60min	126 (80.3)	70 (79.5)	56 (81.2)	
≥60min	31 (19.7)	18 (20.5)	13 (18.8)	n.s. (0.960)

Numbers are *n* (%) reported for valid cases.

ISCED, international standard classification of education; n.s., not significant.

### 3.2 Utilization of digital ICT, online activities in daily life and health related information search

The presence of an internet connection at home (78.4%) and a smartphone (82.6%) was widespread among the study participants. 47.2% of the patients owned a tablet and 11.8% owned a smartwatch. Internet usage was very important in private life for 61.3%. The most important usage could be shown in 57.1% for online news, followed by online shopping (50.9%), online banking (47.8%), and travel bookings (34.2%). 55.4% used the internet for these purposes daily. Social media was used by only 27.2% of the respondents and fitness apps by 16.1%. Overall, the investigated cancer patients were interested in searching health-related information online (71.9%). By far the most significant was the information search about specific diseases (53.1%). This was followed by the online information search about medications, including their effects and side effects (40.0%) and treatment methods (38.8%). Information about nutrition was sought online by 30.6% and information about a healthy lifestyle by 19.4% of the study participants. The online search for hospital and physician rankings (13.8%) and information search about patients’ rights (9.4%) showed the least relevance. Younger [OR 2.42 95%-CI (1.15–5.07)] and higher-educated patients [OR 2.88 (1.26–6.60)], as well as male study participants [OR 2.35 (1.10–5.04)], exhibited a greater affinity for searching for health-related information online. For the internet usage in daily life, a significant association could only be demonstrated for age [OR 8.01 (2.61–24.59)]. There were no significant differences regarding community size and kind of tumor therapy. The results of the multiple logistic regression analysis are shown in detail in Table 2.

**Table 2:** Association between internet usage for daily life activities or for health reasons and age, gender, education level, community size and type of therapy.

	Internet usage for daily life activities	Internet usage for health reasons
	Adjusted OR [95% CI]	Adjusted OR [95% CI]
Age		
≥65 years	1	1
<65 years	**8.01 [2.61–24.59]**	**2.42 [1.15–5.07]**
Gender		
Female	1	1
Male	2.77 [0.98–7.79]	**2.35 [1.10–5.04]**
ISCED		
Low/medium (level 0–4)	1	1
High (level 5–8)	1.93 [0.54–6.89]	**2.88 [1.26–6.60]**
Community size (population)		
≤30.000	1	1
>30.000	1.93 [0.58–6.40]	1.03 [0.48–2.21]
Type of therapy		
Immunotherapy	1	1
Combination with chemotherapy	0.79 [0.20–3.09]	0.35 [0.11–1.09]

Bold values are statistically significant with *p* < 0.05.

OR, odds ratio; CI, confidence interval; ISCED, international standard classification of education.

**Figure 1:**
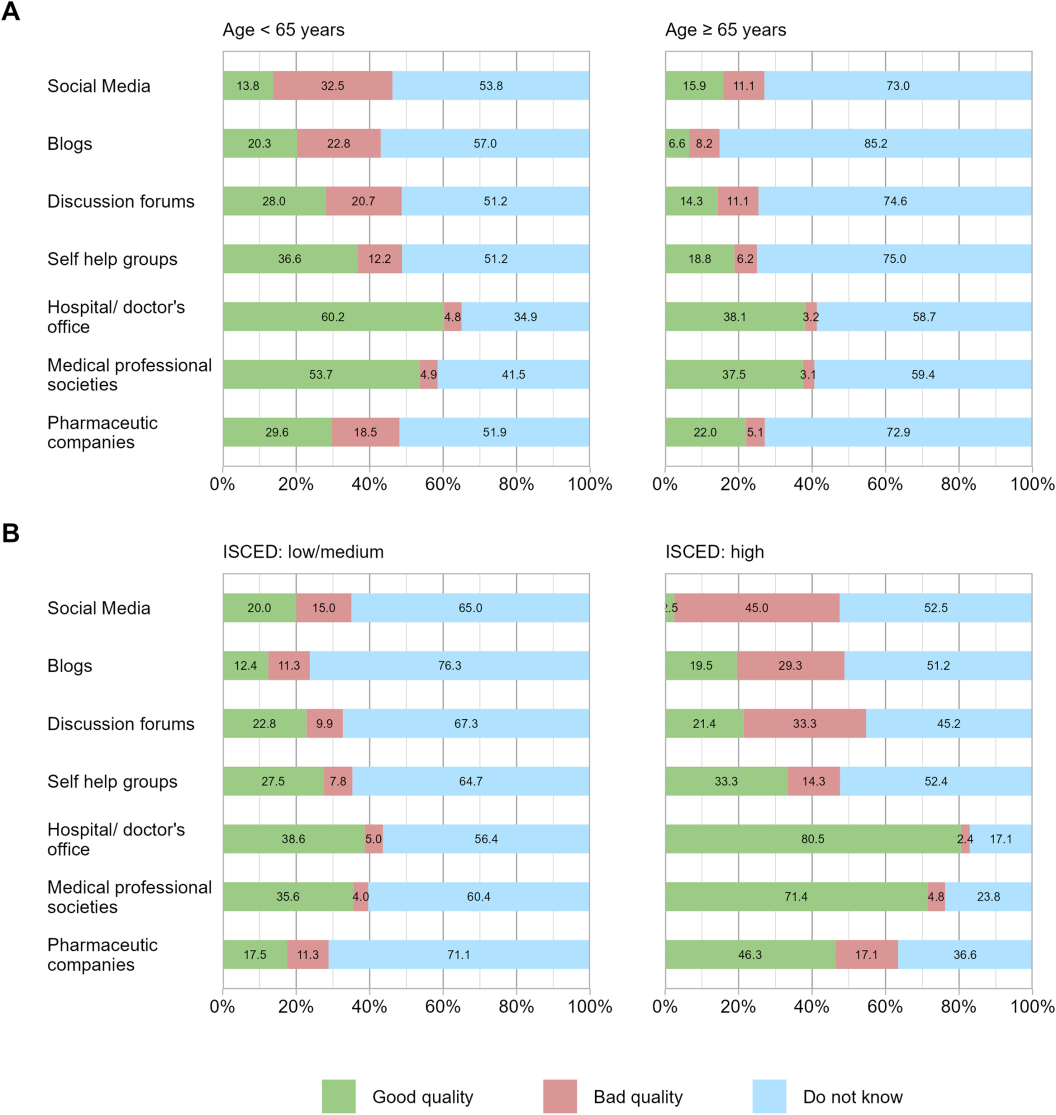
Assessment of the quality of online health-related information sources **(A)** by age and **(B)** by education level. Shown is the assessment of the quality of online health-related information sources according to **(A)** age under 65 and equal/over 65 and **(B)** “medium to low” and “high” education level.

When it comes to the information sources, the cancer patients most often requested online information from the hospitals’ and physicians’ websites (42.1%), followed by medical societies (37.1%), pharmaceutical companies (18.9%), online information from self-help groups (17.7%), discussion forums (14.5%), blogs (11.3%) and social media (10.2%). Analyzing age and educational level, the use of hospitals’ and physicians’ websites and blogs were associated with younger age (*p* < 0.05) while the use of hospitals’ and physicians’ websites, medical societies or pharmaceutical companies were related to higher education (*p* < 0.01). Regarding the assessment of the quality of online health-related information sources, the quality of hospitals’ and doctors’ websites was rated the highest with 50.7%, followed by online information from medical societies (46.6%), self-help groups (28.8%), pharmaceutical companies (26.4%), discussion forums (22.1%), and social media (14.7%). The lowest quality of information was rated for blogs (14.3%). Interestingly, the “Do not know” responses regarding the assessment of the quality of online information sources were noticeably high (up to 69.3%). Overall younger patients and patients with higher education level rated the quality of available online health information sources better (*p* < 0.05) and expressed significantly more confidence in their assessment of these information sources (*p* < 0.05). A detailed presentation is shown in Figure 1.

### 3.3 eHealth literacy

**Figure 2:**
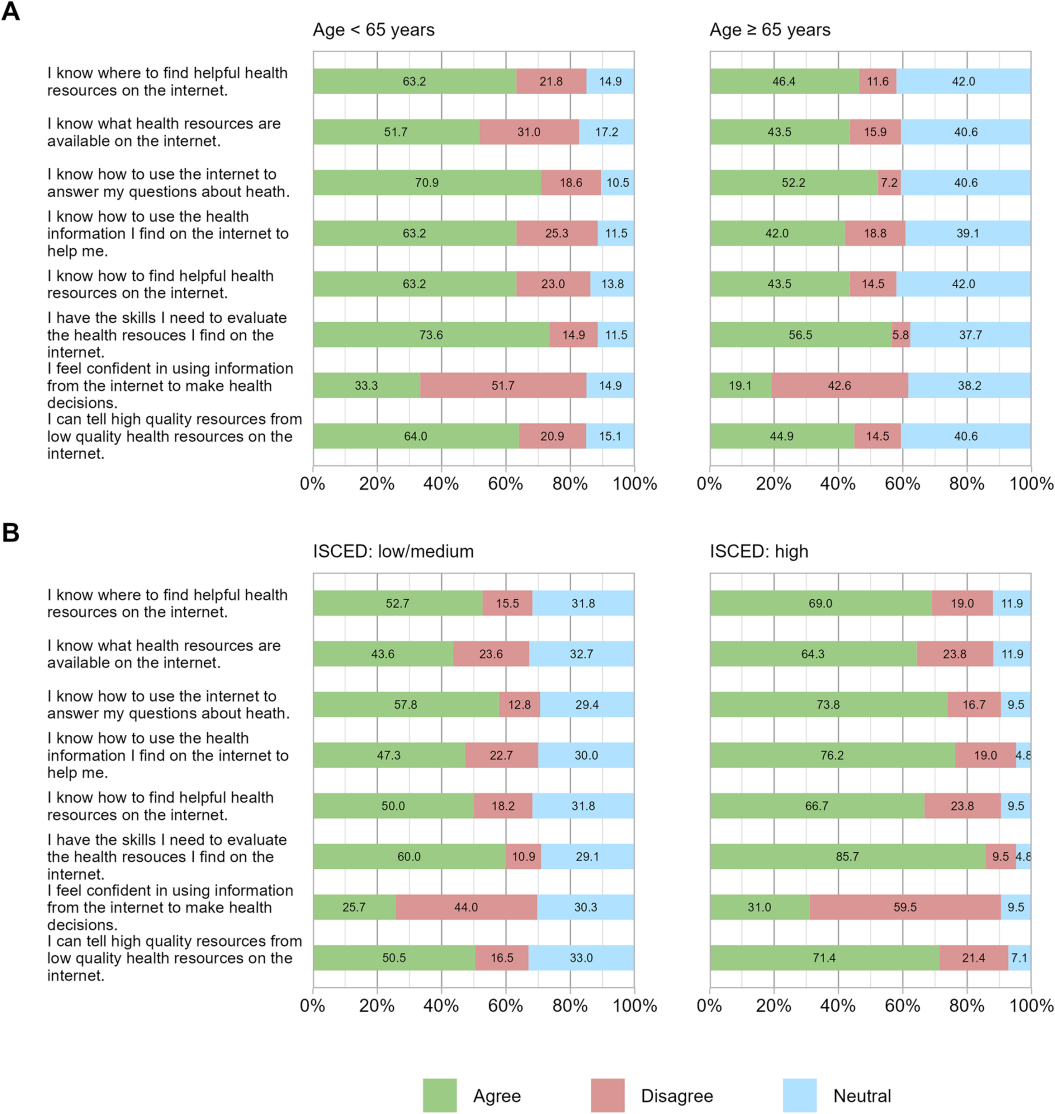
eHealth literacy scale (eHEALS) in percentage (%) by age **(A)** and education level **(B)**. eHealth literacy scale responses are shown categorized in age and education levels: **(A)** age under 65 and equal/over 65 and **(B)** “medium to low” and “high” education level.

In dealing with digital information and communication technologies for health purposes, the patients predominantly indicated that they know how to use the internet for health purposes and evaluate the available online information. Overall, 66.0% responded that they have the skills they need to evaluate the health resources they find on the internet. This positive response was particularly seen in younger (73.6%) and better educated (85.7%) patients as compared to older (56.5%) and less educated (60.0%) study participants. The question “I know what health resources are available on the internet” was answered by 48.1% of the responders with “yes”. Interestingly, only 27.1% of the patients feel confident in using information from the internet to make health decisions. The results of the surveyed questions on eHealth literacy are presented in detail by age and educational level in Figure 2 and show, that in general an overall high eHealth literacy was observed. However, it is still significantly reduced in the older and less educated patient population (highest *p*-value observed between those groups was *p* = 0.037). No differences were seen affecting the type of tumor (lowest *p*-value between type of tumor groups was 0.176).

### 3.4 Regular documentation of personal health information within the scope of outpatient immunotherapy

**Figure 3:**
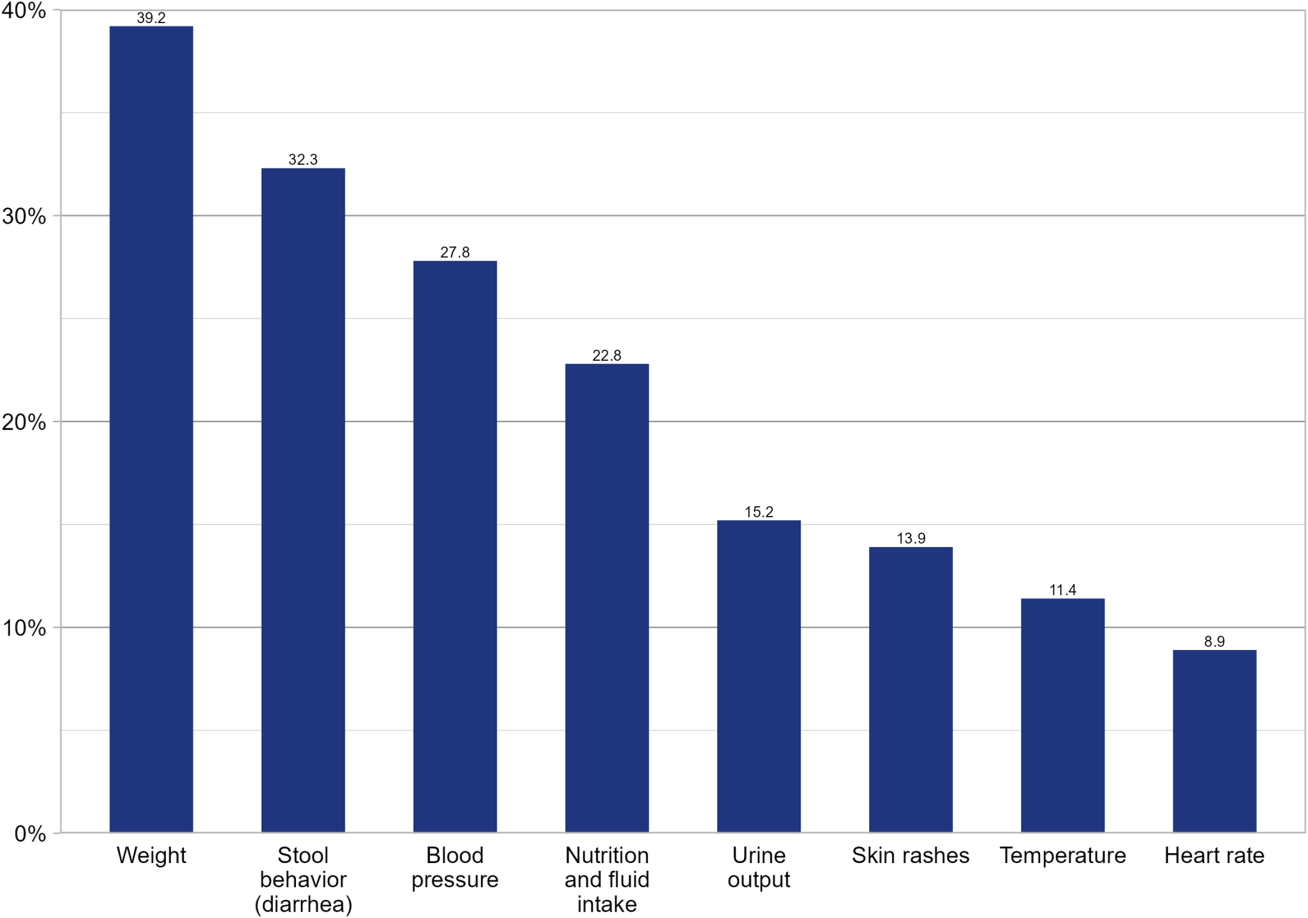
Daily documentation of personal health information within the scope of outpatient immunotherapy. Frequencies of daily documented health parameters are shown in percentage.

The personal health information most often monitored and documented on a regular basis by the study participants was their body weight, with 39.2%, followed by specifics of stool behavior in 32.3% and blood pressure in 27.8%. Body temperature was measured and documented regularly by 11.4%, and pulse rate by 8.9% of the patients. Further details on the nature and extent of daily personal health data collection are presented in Figure 3.

### 3.5 Perspectives of tumor patients on the potentials and concerns of further integration of eHealth applications into outpatient immunotherapy

In summary, the study participants showed a high affinity for the use of eHealth applications in outpatient immunotherapy. Foremost among these was the use of services that are already established in everyday life for appointment booking. The possibility of online appointment scheduling was deemed helpful by 60.6%, automatic appointment reminders via SMS or email by 57.9%, and the automatic delivery of general treatment information (including arrival descriptions) by 59.4%. Also positively evaluated was (1) the ability to digitally communicate examination findings and laboratory results (58.4%), medical reports (60.4%), and medication plans (58.1%), and (2) the use of a hospital app to stay informed about relevant aspects of treatment and exchange information with the hospital (48.4%). The use of online chats with the providers was considered helpful by 39.5%, and the use of video consultations by 34.0%. A locally available treatment option is desired by most of the patients (82.1%). Among all the respondents, 61.0% evaluated the use of online communications between local doctors and healthcare providers at a third level medical center, in the presence of patients, a good way to improve the quality of care in outpatient cancer therapy. Significant differences were observed in the expectations of improving the quality of care through eHealth use in outpatient immunotherapy, with a higher level noted in male [OR 3.30 (1.45–7.49)] and younger [OR 2.63 (1.17–5.90)] patients. Additionally, male patients had a higher affinity towards online appointment scheduling and communication of general information than females [OR 3.51 (1.08–11.37)]. Especially older patients were undecided when answering some of these questions and responded “I don't know” for the questions regarding online appointment scheduling and communication of general information [OR 0.13 (0.03–0.61)], online communication of personal health information [OR 0.13 (0.04–0.48)] and about their attitude towards video consultation [OR 0.40 (0.16–1.00)]. No significant differences were found regarding residential area size, travel time, education level and type of tumor therapy. The detailed results of the multinomial logistic regression analyses are shown in Table 3.

**Table 3:** Association between age, gender and education level with attitudes towards various eHealth usages and the expectations of improving treatment quality through eHealth use.

	Attitude towards online appointment scheduling and communication of general information	Attitude towards online communication of personal health information	Attitude towards online chat and video consultation	Improvement of treatment quality
	Adjusted OR [95%-CI]	Adjusted OR [95%-CI]	Adjusted OR [95%-CI]	Adjusted OR [95%-CI]
Age (ref. ≥65 years)				
<65 years (beneficial)	0.68 [0.20–2.39]	0.79 [0.30–2.04]	1.22 [0.54–2.77]	**2.63 [1.17–5.90]**
<65 years (do not know)	**0.13 [0.03–0.61]**	**0.13 [0.04–0.48]**	**0.40 [0.16–1.00]**	1.35 [0.56–3.27]
Gender (ref. female)				
Male (beneficial)	**3.51 [1.08–11.37]**	1.36 [0.55–3.35]	1.59 [0.70–3.61]	**3.30 [1.45–7.49]**
Male (do not know)	1.34 [0.32–5.70]	0.84 [0.26–2.78]	0.71[0.28–1.79]	**2.79 [1.11–7.05]**
ISCED (ref. low/medium: level 0–4)				
High: level 5–8 (beneficial)	1.50 [0.38–5.93]	0.90 [0.34–2.34]	1.72 [0.71–4.16]	1.13 [0.49–2.61]
High: level 5–8 (do not know)	1.17 [0.22–6.31]	0.25 [0.05–1.14]	0.55 [0.17–1.74]	0.45 [0.15–1.37]

Bold values are statistically significant with *p* < 0.05. Ref., reference level; OR, odds ratio; CI, confidence interval; ISCED, international standard classification of education.

**Figure 4:**
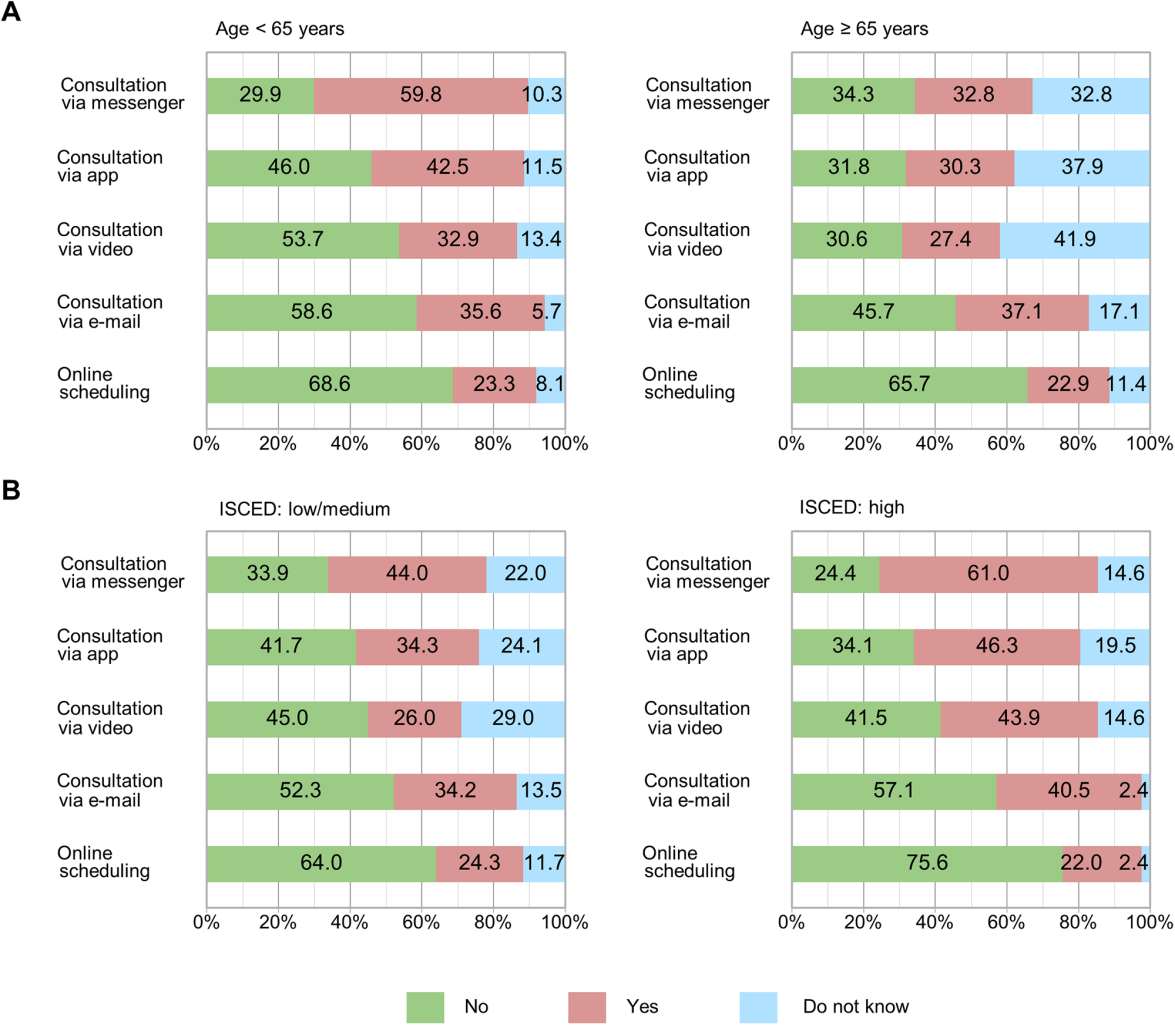
Concerns in data privacy and data security regarding the use of eHealth by type of eHealth service. **(A)** By age and **(B)** by education level. Data security concerns stratified by **(A)** age under 65 and equal/over 65 and **(B)** “medium to low” and “high” education level. Each bar chart represents one of the five distinct eHealth service applications evaluated.

Regarding data privacy and data security, 23.1% of the respondents expressed concerns in administrative processes without the exchange of personal medical information (e.g., online appointment scheduling). With the exchange of personal medical information via email, 36.3% expressed concerns about data security. 37.3% expressed concerns about data privacy and data security when using health apps, 30.6% in video consultations, and 48.1% in personal health data exchange via health messengers. Age-specific significances regarding data security were only observed in the use of health apps, messenger services, and video consultations. Here the older study participants showed fewer concerns overall (highest *p*-value between the age groups: <0.001). No significances were found regarding gender, educational level, residential area, and type of tumor therapy (lowest *p*-value observed between the groups: 0.064). The age-related and education-related details are presented in Figure 4.

## 4 Discussion

Digitization is rapidly advancing in many areas of life, particularly in the healthcare sector (43–46). Alongside the currently very focused developments in artificial intelligence in medicine, various medical fields are showing that the digital information and communication technologies, which are already widely used in private and professional life, increasingly have the potential to be used for cross-location and cross-sectoral communication between healthcare providers and patients (47–49). This is particularly important for oncological immunotherapy. Immunotherapy can increasingly be provided on an outpatient basis with the assurance of home monitoring. And precisely for this purpose, a barrier-free telemedicine connection for patients to a comprehensive care center can be helpful. Despite this undoubtedly great potential of telemedicine, little is known about the use of modern information and communication technologies in daily life in this patient population, especially for their personal health or treatment purposes. Of particular interest for the increasing number of outpatient care patients in oncology is the demographic trend with a significant increase in the proportion of older population groups within the overall population (50, 51). The proportion of young individuals in the total population is decreasing, while the number of predominantly old patients in the field of oncology is increasing (52). In Germany alone, where the current study was conducted, there will be a projected 26% increase in cancer patients according to current forecasts (53), accompanied by a demographically driven reduction in healthcare professionals (54). Possible consequences of these developments include reduced doctor-patient contacts, extended waiting times for treatments and longer travel duration for patients. These trends may lead to lower compliance with tumor patients attending appointments and presenting for treatment of acute complaints (55). One possible approach to adequately address these developments is the increased use of eHealth for cross-sectoral cancer care (48). Nevertheless, usage of eHealth applications, in the context of outpatient therapy for hematological-oncological patients, is neglected (56). Therefore, there is a particular need to better understand the attitudes of cancer patients as well as influencing factors of various aspects of modern ICT usage for private live and for health reasons with a special focus on health-related information search and online communication and information transfer in cross-sectoral oncological immunotherapy care. Our study provides important insights into these aspects as well as the factors that influence the further integration of eHealth applications into outpatient immunotherapy from the patients’ perspective and highlights in which fields target-group specific action is required.

Our study population showed a high penetration of modern ICT with 82.6% owning a smartphone and 78.4% having an internet connection at home. This is in line with previous reports and supports our study patients as a representative cohort (57–59). This is also true for the older mean age of our patients, which is average in many malignant diseases and therefore differs from the mean age of patient populations in eHealth studies outside the field of oncology, focusing on non-malignant diseases (49, 60). Many of them used the internet daily for everyday life activities (55.4%), with more younger patients using it than older patients, whereas gender, education level, community size, or type of treatment did not show significant differences. Although the usage rate is lower than in a recently done study in the field of otolaryngology, the age dependency aligns with the findings of this study and can explain the generally lower usage rate (49). Based on our findings, when it comes to modern ICT and internet use for health reasons, however, not only age was a significant factor, but also males and patients with higher education levels showed stronger online health information seeking (OHIS) behavior than females or patients with lower education levels. These findings are in line with previous studies carried out in Germany in different medical fields and within the general population, expect for the higher affinity of men for OHIS (49, 60, 61). Our study shows that the main topics searched for were information on specific diseases, medications, and treatment options, which is consistent with the OHIS behavior of patients in the field of otolaryngology (49). While the differing results in gender in our analysis cannot be ultimately explained, it must be noted that the underlying disease and the treatment methods might also play essential roles in OHIS and should be further investigated, especially potential differences in patients with acute and chronic diseases as well as non-malignant and malignant diseases. Furthermore, our findings support the hypothesis that OHIS has a central role in individual patient education and will become increasingly important for patient's proficiency and empowerment. Therefore, a closer look at the online-available health-related information sources and their assessment by oncology patients is becoming increasingly important. Our study showed that for the study population of oncology patients, the most trusted online sources for health information were hospitals/physicians’ websites and online information from medical societies whereas the least trusted sources were social media and blogs. The great importance of information provided online by medical experts is consistent with a recent study in the field of otolaryngology, which shows that 77.9% of patients demand approval of medical information by professionals (49). Especially younger or well-educated patients showed in our patient cohort more self-confidence in using and selecting between well-accepted and rejected online sources for OHIS. Interestingly, the older and less educated patient population was more likely to show indecision when answering these questions. These results indicate a special need for a better and especially targeted group-oriented promotion of patient education in the environment with these online information sources. Furthermore, these findings highlight the need for increased quality checks by medical oncology experts and official recommendations for trusted sources of information for oncology patients. Our study also shows that 27.2% of the surveyed patients indicated regular use of social media. This demonstrates the potential of these digital platforms for the utilization of online support groups especially for younger patients and their relatives in the field of oncology. Even though the usage of fitness apps within the examined oncology patient population is currently low at 16.1%, this aspect should be continuously monitored. This need is underscored by the requirement of close monitoring of various vital parameters and other personal health information due to possible severe immune-related adverse events especially in patients undergoing outpatient immunotherapy with ICI. A regular screening for these symptoms and rapid identification of a possible treatment indication in the home environment is essential for patient safety and this can be supported by the use of modern ICT by patients and caregivers. We are convinced that the adoption of these devices in the oncology patient population will continue to increase rapidly, allowing smartwatches, fitness trackers and further cloud-based eHealth applications with modern interfaces to be more frequently integrated into mobile care in the field of oncology in the near future.

Another important aspect is the eHealth literacy of oncology patients undergoing immunotherapy. eHealth literacy hereby refers to the measure of how patients utilize digital information sources to solve a health issue (62). Patients with higher eHealth literacy are not only more likely to use modern information and communication technologies for health reasons but are also more skilled in understanding and utilizing the digital resources found (63). The present study results demonstrate that while the eHealth literacy of the examined patient population is generally high, there is still a significant difference in terms of age and educational level. Younger and generally better-educated patients undergoing oncological immunotherapy can therefore utilize modern information and communication technologies more effectively to solve their health problems and engage in their own health prevention, as has been demonstrated in various previous studies involving chronically ill patients (64). Patients with low eHealth literacy do not fundamentally reject eHealth but rather appear inexperienced and therefore uncertain in their usage, this underscores the significant need for targeted group-specific educational methods for utilizing modern information and communication technologies for oncology patients and, from the authors’ perspective, for the entire population to enhance prevention, diagnosis, therapy, and follow-up care. Assistance through eHealth applications is a very promising option to overcome this impediment, for instance via regular online consultations with the specialist or an automated alarm system notifying the physicians via app, when symptoms occur. Overall, our patient cohort is in favor for telemedical support during their treatment and finds it benefitting for the treatment quality. This is not only true for automated app-based contacting with the treatment center, but also for online consultations and administrative tasks such as online scheduling and receiving medical test results, discharge summaries and medication plans online. Especially the younger and male patient population expected that a deeper integration of eHealth in outpatient immunotherapy can improve the quality of care. Although not the entire patient population is equally convinced of the benefits of using digital ICT for their own health, this nonetheless provides a good starting point for increased utilization of these media in routine healthcare. Very important for further integration of eHealth applications into the outpatient care of patients undergoing oncological immunotherapy is the consideration of data security and data privacy. Our study results indicate that this is very important not only from the health authoritieś but also from the patientś perspective, especially when it comes to online communication of sensitive personal health information. The concerns of patients regarding data privacy and data security furthermore showed a dependence on the type of eHealth application involved. However, over 60% of the study population expressed that the use of eHealth applications would improve the treatment quality in oncological outpatient immunotherapy. This underscores the significant potential for further integration of eHealth applications to overcome the boundaries of space and time and enhance outpatient oncologic immunotherapy. From the perspective of the investigated oncological patient population, this potential is markedly greater than in a previously published patient collective in the field of otolaryngology (49). Although a direct statistical comparison between the previous study and our current results is not possible, the descriptive data suggest that oncology patients undergoing recurring outpatient immunotherapy have higher expectations for the use of eHealth applications to improve treatment quality (over 60% in the present study compared to 21.2% in the previous study). Additionally, although not directly comparable, the data indicate lower concerns about data security in the current patient population compared to the study population investigated in the field of otolaryngology, which did not focus on malignant diseases and had a markedly lower average age. In that study, 64.6% of patients expressed concerns about data security (49). From the authors’ perspective, this underscores the need for a medically and technologically controlled, secured, and scientifically investigated integration of eHealth applications in outpatient oncology care, as well as the in-depth investigation of the different perspectives of various patient populations on data security, data privacy, and demand-oriented data availability. At the same time, the expressed concerns regarding data security and privacy highlight the necessity for very high standards of data protection and security when using modern ICT for digital care pathways in outpatient immunotherapy. This need remains paramount, even though the concerns about data security were expressed to a lesser degree than by the patient population in the field of otolaryngology (49). Due to the already existing uniform legal regulations and required high data security and privacy standards for eHealth applications in general, the necessity for better patient education is particularly evident for this thematic focus, also to earn trust from the patients for new cross-location digital care pathways.

This study also supports that it is important to design target-group specific eHealth applications and to implement innovative learning methods to increase digital competence and eHealth literacy in patients. This is strong data to support that telemedicine and eHealth literacy need to adapt to individual needs. Access to eHealth applications must be possible without barriers, which is in line with findings of recent eHealth studies outside the field of outpatient immunotherapy (49, 65). Before the target-group specific eHealth applications can be used, patients must be individually educated and the right handling must be exercised. The trainings should be provided and regular check-ups must be performed with standardized interim evaluations (21). As digital ICT is used adjunct to immunotherapy, physicians need to have a certain level of knowledge and should also undergo training before working with eHealth applications (21). Inconsistencies in the usage of eHealth applications could have a negative impact on patients’ compliance and on the motivation of the treating physicians. That targeted media development is required was also shown by Brew-Sam et al., who compared 121 applications for diabetes’ management, concluding that mobile apps for diabetes’ self-management are not able to provide relevant features for empowering patients. One big reason is the lack of providing an opportunity to individualize and adapt the applications. Most of the apps failed to tailor services to specific patient subgroups with differing needs (66). One reason might be that many eHealth applications are developed by young digital savvy and healthy people without really understanding the way to really improve cross-sectoral digital care pathways (67). Therefore, the authors of the present study recommend that specific eHealth applications in the field of oncology and especially in oncologic immunotherapy should be developed not only by information technology savvy people but also by interdisciplinary and multiprofessional teams consisting of patients, oncologists, care managers, nurses and furthermore experts in data privacy and data security.

In addition to the further need of personalized and individualized healthcare applications for patient groups, the quality of the applications must also be guaranteed. In addition to “telemedicine” and “eHealth”, the WHO also defines a mobile component, “mobile health (mhealth)”. This refers to the use of any type of mobile device, e.g., through the use of smartphones, tablets, wearables, etc., in the context of healthcare (68). Statista showed that in 2020 around 48,608 medical mobile Health apps were available to download from the Apple App Store worldwide (69). This underlines the huge market for health apps, but the availability of quality approved apps that can be recommended for treatment and therefore prescribed by doctors is minimal. Additionally, the German consumer advice organization reported that some of the apps provided are of insufficient scientific value and may even cause harm (70). These data show that next to individual patient education and tailored development of eHealth applications, there is also a need for further scientific verification and regular review of medical applications and devices with a special focus on quality, efficacy, efficiency and safety.

This is also inevitable to alleviate patients’ concerns about data privacy and security, which are seen in our patient cohort as well. Trust and security are essential to promote the required compliance for the use of eHealth media. The global use of ICT is very high: The volume of data generated or replicated worldwide was 64.2 zettabytes in 2020. The forecast for 2025 is 181 zettabytes (71). The number of e-mails sent worldwide every day was 333.2 billion in 2022 (72). 667 million “WhatsApp”-Messages were sent per day in Germany in 2015 (73). In addition, private information is shared in a variety of other ways: The Federal Statistical Office in Germany reported that 59% of the German population (between 16 and 74 years) used online banking (74). Studies showed that 50% of the German-speaking population aged 14 and over used social media at least once a week (75) and in the first quarter of 2023, 5.84 billion payment transactions were processed via PayPal (76). In light of these examples, it becomes apparent that data security concerns in patients differ between private information and highly sensitive information like health-related issues. These results make clear that, on one hand, more detailed research is needed into the composition of patients’ concerns about data privacy and security, and on the other hand a generally applicable quality label also ensuring data safety urgently needs to be implemented. Development of standard regulations with detailed scientific testing that is made visible through uniform quality labels might be a good way to gain further trust in patients using medical apps and devices (77).

Overall, our data show a high distribution of internet-enabled devices and generally a positive attitude towards eHealth in oncological patients undergoing immunotherapy with ICI. This is particularly interesting since these patients need close monitoring in the outpatient setting due to possible acute and severe immune-related toxicities. Telemedical facilitation of immunotherapy treatments is likely to substantially increase patient safety and treatment benefit (78–80). With this, the results from our analysis could also serve as indicators of the potential of eHealth in patients undergoing other forms of immunotherapy such as treatment with bispecific antibodies or with cellular immunotherapies such as chimeric antigen receptor (CAR) T-cell therapies or allogeneic hematopoietic stem cell transplantation (allo-HSCT), which require close monitoring and early intervention, if complications are suspected. Patients following an allo-HSCT can develop severe transplant-related life-threatening acute complications including infections (81) and *Graft-*vs.*-Host Disease* for a prolonged time-period (82–84). The outpatient follow-up of these patients is therefore bounded to a specialized transplant center. Implementation of eHealth options in allo-HSCT patient aftercare might not only reduce patient visits to the transplant center with often long travel times, but also help to detect possible life-threatening complications early in the home environment enabling rapid intervention and therefore increasing patient safety dramatically (85). CAR T-cell treated patients can develop treatment-related toxicities such as cytokine release syndrome (86) or immune-cell associated neurotoxicity syndrome (87). These can be fatal and thus, these therapies are performed as an inpatient in most cases. With more experience with CAR T-cell therapies in many centers, to alleviate the treatment burden for the patients and to ensure capacity for all patients with more indications approved and increasing numbers of CAR T patients expected, the urge to perform CAR T-cell therapies in the outpatient setting is growing (88). For these patients, eHealth support would also be highly feasible to increase patient safety (89). Nevertheless, detailed assessment of the potential of eHealth in patients undergoing allo-HSCT or CAR T-cell therapies is urgently needed to be able to identify the best treatment- and patient-tailored eHealth options.

We acknowledge several limitations in this study, which must be considered when interpreting the results. While the cognitive condition of the patients was assessed during patient-physician interaction and through detailed review of the medical history, no specific cognitive screening tool was used. Additionally, the questionnaire was extensive. Many of the patients undergoing active therapy are seriously ill and therefore it was sometimes difficult to answer all the questions for some patients. Moreover, an assessment of differences considering the treatment duration with ICI at the time of answering the questionnaire was not evaluated in detail. The duration of ICI administration as well as the treatment plan could potentially bias the patients’ answers and need to be incorporated in future studies. This is also true for considering the type of cancer and administration setting. Lastly, and most importantly, our questionnaire did not include assessment of the patients’ view on the use of artificial intelligence in healthcare. This field is becoming increasingly important in eHealth and needs to be urgently addressed in future studies. Nevertheless, our results lay an excellent foundation for further digitalization of cross-sectoral care in the field of immunotherapy.

In conclusion, our study shows a generally high acceptance of eHealth in patients undergoing outpatient immunotherapy with ICI. It supports the huge potential of telemedicine in this patient cohort and forms the basis for the development of treatment- and patient-tailored cross-sectoral eHealth platforms. However, there is no “one-answer-fits-all” solution. Especially the varying views on particular aspects of eHealth by specific patient subpopulations, for example by age, education level, gender and others, strongly point to the need of designing patient-specific telemedical options and patient-centered eHealth training and education. Explicitly focusing on patients undergoing immunotherapies with specific potential toxicities and outpatient management, the addition of eHealth applications could be feasible. Further studies are needed in oncological immunotherapy patients, including cellular immunotherapies like allo-HSCT or CAR-T-cell therapies, to assess the special needs of patient subgroups for the development of customized eHealth options and educational methods.

## Data Availability

The raw data supporting the conclusions of this article will be made available by the authors, without undue reservation.
